# Cervical cancer burden among females under 40 years in China, Japan, and South Korea, 1990–2021: a systematic analysis for the global burden of disease study 2021

**DOI:** 10.3389/fpubh.2025.1695004

**Published:** 2025-11-13

**Authors:** Caiyan Xiao, Juan Deng, Fangyuan Ren, Chan Zhang

**Affiliations:** Department of Gynaecology and Obstetrics, The Affiliated Zhuzhou Hospital Xiangya Medical College, Central South University, Zhuzhou, China

**Keywords:** cervical cancer, global burden of disease, East Asia, epidemiology, public health

## Abstract

**Background:**

Cervical cancer remains a major global public health concern; however, its burden among younger women in East Asia has not been systematically characterized across spatial, temporal, and age dimensions.

**Methods:**

This study analyzed the incidence, prevalence, mortality, and DALYs of cervical cancer among females aged <40 years in China, Japan, and South Korea from 1990 to 2021 using GBD 2021 data. The results highlight distinct geographic and temporal patterns with urgent implications for public health policies. Strengthening HPV vaccination, expanding screening, and targeted campaigns are critical to reducing the burden and aligning with the WHO elimination goals.

**Results:**

Between 1990 and 2021, the burden of cervical cancer among females aged <40 years in China, Japan, and South Korea showed distinct geographic and temporal patterns. In 2021, Japan maintained the highest incidence, prevalence, and DALYs, whereas China recorded the highest mortality. Temporal trends indicated a rising incidence in Japan and China but a decline in South Korea, while mortality and DALY rates decreased substantially in China and South Korea and remained low in Japan. Across all countries, the burden was predominantly concentrated in women aged 30–39 years, with a stable overall age composition, but a slight increase in the proportion of the 35–39-year group over time.

**Conclusion:**

Despite modest reductions in age-standardized burden, cervical cancer remains a significant issue for women under 40 years of age in East Asia, compounded by suboptimal HPV vaccine uptake and COVID-era disruptions. Therefore, strengthening vaccination, expanding screening, and launching public health campaigns are urgent priorities.

## Introduction

Cervical cancer ranks among the most significant female malignancies globally, especially in low- and middle-income settings, where preventive measures such as organized screening and vaccination remain suboptimal. According to the World Health Organization (WHO), cervical cancer is the fourth most common cancer and the fourth leading cause of cancer-related deaths in women worldwide in 2020, with an estimated 604,000 new cases and 342,000 deaths (1). Despite HPV vaccination and effective screening being preventable, a disproportionate burden persists in regions with limited access to such interventions.

The Asia–Oceania region accounts for over half of the global cervical cancer cases and deaths, highlighting the urgency of addressing this burden (2). Within this region, East Asia, comprising China, Japan, and South Korea, shows marked heterogeneity in cervical cancer epidemiology and public health policies. Although age-standardized incidence and mortality rates have declined in countries with high SDI, disparities persist across nations with differing development levels. Importantly, the burden on women under 40 years of age remains understudied, leaving a critical gap in targeted prevention strategies (3).

GBD 2021 analyses show that across Asia, higher SDI correlates with lower cervical cancer burden: age-standardized mortality rate (ASMR) and age-standardized DALY rate (ASDR) display significant negative correlations with sexual disease infection (SDI) (*r* = −0.49 and *r* = −0.53, respectively), while ASIR (age-standardized incidence rate) was also negatively correlated, although not significantly (3). This highlights the protective influence of socioeconomic development and robust health systems on cervical cancer.

Focusing on younger women, those under 40 years of age are particularly important. The 15–39 age group, or the broader reproductive age range, bears substantial morbidity affecting fertility, economic productivity, and quality of life. In Japan, cervical cancer is the most prevalent cancer among women aged 15–39 (4). However, epidemiological studies stratified for this demographic in East Asia remain limited, hindering the development of precise targeted prevention strategies.

HPV vaccination, the cornerstone of primary prevention, has been unevenly adopted across East Asia. Variations in HPV vaccination policies across East Asia have significantly influenced cervical cancer prevention. In China, HPV vaccines have been available since 2016, but coverage remains low and often out-of-pocket, with limited public funding or inclusion in national immunization programs (1, 5). Japan’s experience illustrates the fragility of public trust: HPV vaccination was included in routine immunization in 2013, but active government recommendations were suspended soon after, leading to a fall from about 70% to under 1% (4). Although recommendations resumed in 2021 and coverage began to recover (~30% first-dose uptake in 2022) (4), vaccination rates remain well below the WHO target of 90% by the age of 15 (6). In contrast, South Korea introduced HPV vaccination into its national immunization schedule in 2016, achieving a relatively higher coverage (~87% first-dose uptake in target age groups) (7, 8). These divergent experiences highlight the critical impact of policy decisions on vaccination uptake and cervical cancer burden, thus forming a key rationale for this study.

However, secondary prevention through screening remains unexplored. Many Asia–Oceania countries rely on opportunistic screening with variable quality and coverage (2), which undermines effectiveness, particularly in populations that lack consistent healthcare access.

Given the preventable nature of cervical cancer, the WHO’s global elimination strategy has set ambitious 2030 targets: 90% of girls vaccinated by age 15, 70% of women screened with high-quality tests by ages 35 and 45, and 90% of women with cervical disease receiving treatment (6). Achieving these targets depends on closing gaps in both vaccination and screening.

Using GBD 2021 data, this study aimed to quantify the burden of cervical cancer in females under 40 years of age in China, Japan, and South Korea, characterizing trends from 1990 to 2021 and examining disparities in relation to SDI, vaccination, and screening. We focus on this younger demographic, given their critical developmental and reproductive roles and the potential for impactful intervention during early adulthood. Through this, we aim to develop tailored public health strategies aligned with the WHO’s elimination goals and the East Asian context.

## Methods

### Data source

This study was designed as a population-based, descriptive epidemiological analysis using publicly available data from the Global Burden of Disease Study 2021 (GBD 2021), developed by the Institute for Health Metrics and Evaluation (IHME) (9). The GBD 2021 database provides annual estimates of the disease burden for 369 diseases and injuries across 204 countries and territories from 1990 to 2021, including cervical cancer (ICD-10 code C53), as defined by the International Classification of Diseases (ICD), Tenth Revision. Data were accessed using the GBD Results Tool,^1^ and country-level estimates were extracted for China, Japan, and South Korea.^2^

All estimates in this study are derived from the modeled outputs of the GBD 2021 framework, which synthesizes registry, survey, and vital statistics data through standardized statistical modeling. Therefore, the trends represent modeled estimates rather than direct registry observations.

### Study population and variables

The study population comprised all females aged <40 years residing in China, Japan, and South Korea between 1990 and 2021. Demographic data (population denominators) for the rate calculations were obtained directly from the GBD database, which applies a standardized, internally consistent set of population estimates derived from censuses, surveys, and vital registration systems. This study focused exclusively on females under 40 years of age, stratified into the following age groups: <20, 20–24, 25–29, 30–34, and 35–39 years. For females under 40 years, data were extracted in 5-year age strata (<20, 20–24, 25–29, 30–34, 35–39 years) from the GBD Results Tool. When estimates were only available for broader age bands (e.g., 15–49 years), population-weighted interpolation was applied using IHME’s standard population dataset to derive consistent estimates for the target group. This ensured internal comparability while preserving the original GBD age-standardization framework. The extracted variables included incidence, prevalence, mortality, and disability-adjusted life years (DALYs), which collectively enabled a multidimensional assessment of cervical cancer burden and facilitated both cross-regional and temporal comparisons.

### Estimated annual percentage change analysis

The estimated annual percentage change (EAPC) was calculated to assess temporal trends in incidence rates and DALYs. Based on a log-linear regression model, EAPC is well suited for detecting changes in epidemiological trends over time, providing a robust method for analyzing the evolution of cervical cancer burden in our study population. It represents the average annual percentage change over a specified period, and was calculated using the following formula, where *β* denotes the regression slope:


$$\text{EAPC}=(e^{\beta }-1)*100$$

EAPC provides a robust measure of the average annual percentage change over a specified period, while Joinpoint regression helps identify significant changes in trends. Temporal trend analyses were conducted using the Joinpoint Regression Program (version 4.9.1.0, National Cancer Institute, USA). Up to three joinpoints were allowed, and Monte Carlo permutation tests (significance threshold *p* < 0.05) were used to identify statistically significant changes in slope. When no significant joinpoints were detected, a single-segment log-linear model was applied to estimate the overall EAPC.

### Geospatial visualization of cervical cancer burden

To illustrate the geographical distribution of cervical cancer burden among females under 40 years of age in the three East Asian countries, geospatial maps were generated using epidemiological indicators, including incidence, prevalence, mortality, and DALYs. The data for these indicators were obtained from the GBD 2021 study. Maps were produced using the R software, with color gradations applied to represent the relative levels of disease burden across countries.

### Trend analysis and visualization

To depict temporal trends in cervical cancer burden among females under 40 years of age in China, Japan, and South Korea, annual incidence, prevalence, mortality, and DALY rates from 1990 to 2021 were compiled. Stacked bar charts were used to illustrate the annual distribution across countries, and smoothed trend curves were applied to visualize long-term changes. Data processing and visualization were conducted using the R software.

### Data analysis and software

All statistical analyses were performed using R software (version 4.1.2) and the Joinpoint Regression Program (version 4.9.1.0) of the U.S. National Cancer Institute (10). The R packages used included the data table, dplyr, Epi, ggplot2, ggsci, magrittr, RColorBrewer, readxl, factoextra, openxlsx, purrr, tidyr, ggpubr, ggrepel, parallel, broom, car, MASS, mgcv., splines, ggmap, tidyverse, maps, rgdal, reshape, and forecast. Graphs were generated to visualize temporal trends, country comparisons, and risk factor contributions. All *p* values were two-sided, and statistical significance was set at *p* < 0.05.

## Results

### Geographical distribution of cervical cancer burden

Between 1990 and 2021, the age-specific incidence and DALY rates of cervical cancer among females under 40 years of age showed marked variation across countries (Table 1). In China, the incidence increased in all age groups over 20 years, with the greatest rises in women aged 25–29 years (EAPC = 1.24; 95% CI: 1.04–1.25) and 30–34 years (EAPC = 1.23; 95% CI: 0.95–1.31), while the <20-year group demonstrated a declining trend (EAPC = −0.38; 95% CI: −0.52 to −0.29). The DALY rates decreased across all age groups, with the largest reduction in the <20-year group (EAPC = −3.14; 95% CI: −3.26, −3.07).

**Table 1:** Estimated annual percentage change (EAPC) in rates of incidence and DALYs of cervical cancer among females under 40 years old in China, Japan, Republic of Korea, and globally, 1990–2021 (with 95% CI).

Age/location	Incidence				DALYs			
	China	Japan	South Korea	Global	China	Japan	South Korea	Global
<20 years	−0.38 (−0.52, −0.29)	−0.27 (−0.45, −0.16)	−0.83 (−1.07, −0.58)	−0.00 (−0.08, 0.36)	−3.14 (−3.26, −3.07)	−1.74 (−1.74, −1.70)	−3.69 (−3.95, −3.36)	−0.84 (−0.92, −0.53)
20–24 years	0.80 (0.68, 0.84)	0.69 (0.55, 0.81)	−0.21 (−0.41, 0.08)	0.07 (−0.00, 0.28)	−2.03 (−2.14, −1.93)	−0.80 (−0.81, −0.79)	−3.09 (−3.30, −2.88)	−0.70 (−0.78, −0.51)
25–29 years	1.24 (1.04, 1.25)	1.11 (0.97, 1.22)	0.03 (−0.17, 0.34)	−0.08 (−0.14, −0.00)	−1.63 (−1.82, −1.60)	−0.36 (−0.39, −0.31)	−2.81 (−3.03, −2.42)	−0.83 (−0.87, −0.77)
30–34 years	1.23 (0.95, 1.31)	1.98 (1.87, 2.08)	−0.06 (−0.26, 0.24)	−0.33 (−0.42, −0.20)	−1.53 (−1.79, −1.43)	0.77 (0.74, 0.82)	−2.77 (−2.95, −2.45)	−1.14 (−1.21, −1.03)
35–39 years	1.07 (0.71, 1.22)	1.96 (1.82, 2.09)	−0.86 (−1.12, −0.64)	−0.42 (−0.49, −0.33)	−1.49 (−1.83, −1.34)	1.06 (1.05, 1.09)	−3.39 (−3.58, −3.21)	−1.23 (−1.27, −1.16)

95% CI, 95% Confidence Intervals.

In Japan, incidence rose in all age groups ≥20 years, with the steepest increases in the 30–34 years (EAPC = 1.98; 95% CI: 1.87–2.08) and 35–39 years (EAPC = 1.96; 95% CI: 1.82–2.09) cohorts. DALY rates declined in women under 30 years but increased in those aged ≥30 years, particularly in the 35–39-year group (EAPC = 1.06; 95% CI: 1.05–1.09).

In South Korea, the incidence generally declined, most prominently in the <20-year (EAPC = −0.83; 95% CI: −1.07 to −0.58) and 35–39-year (EAPC = −0.86; 95% CI: −1.12, −0.64) groups, accompanied by marked reductions in DALY rates across all age groups, with the largest decrease in the <20-year group (EAPC = −3.69; 95% CI: −3.95, −3.36).

Globally, the incidence remained stable or slightly decreased in most age groups, whereas DALY rates declined across all groups, with the steepest reduction in the 35–39-year group (EAPC = −1.23; 95% CI: −1.27 to −1.16).

**Figure 1:**
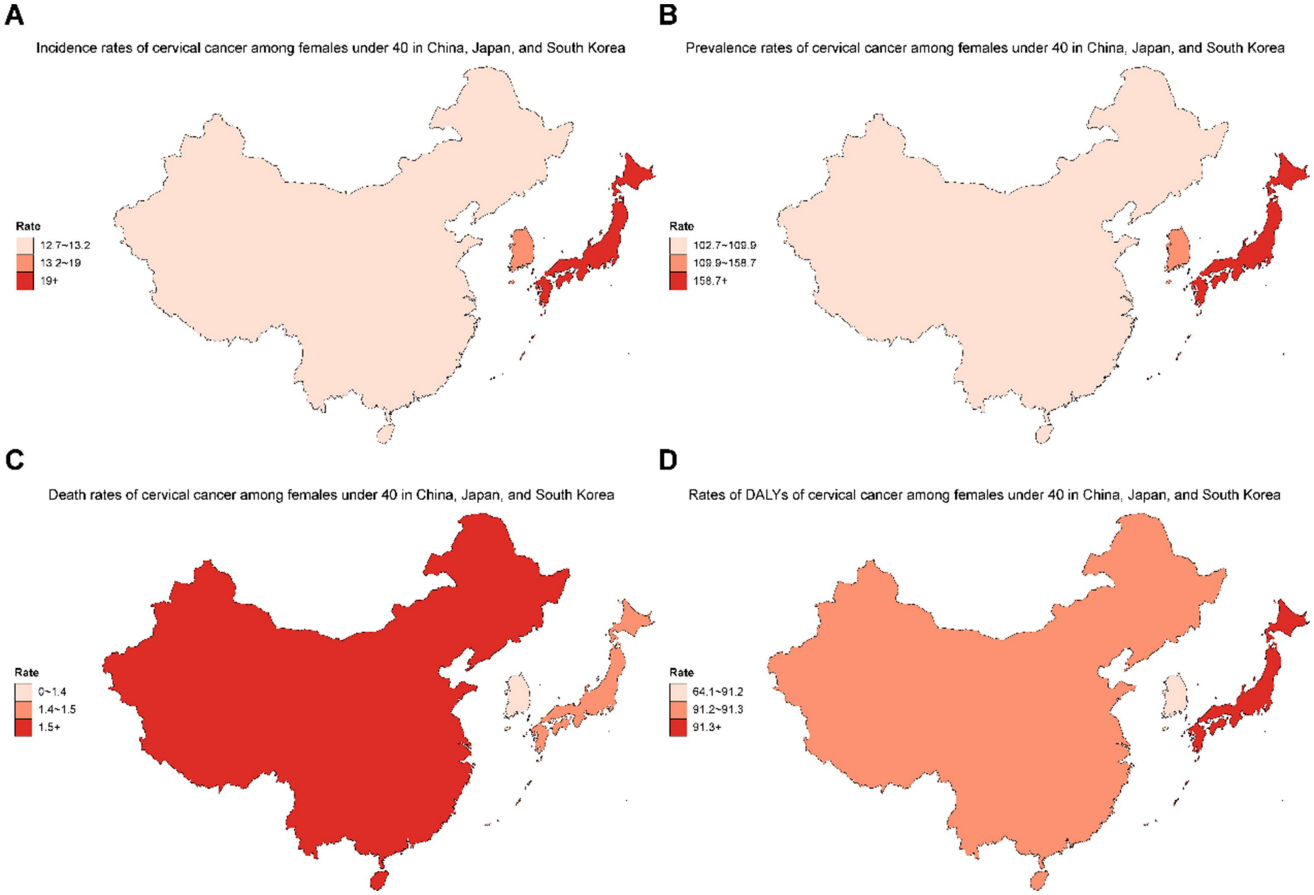
Geographical distribution of incidence **(A)**, prevalence **(B)**, death **(C)**, and DALYs **(D)** of cervical cancer among females under 40 years old in China, Japan, and South Korea in 2021.

In 2021, the geographical distribution of the cervical cancer burden among females under 40 years of age varied substantially across the three countries (Figure 1). Japan exhibited the highest incidence (≥19), prevalence (≥158.7), and DALY rate (≥91.3), while China had the highest mortality rate (≥1.5). South Korea demonstrated comparatively lower rates for all four indicators.

**Figure 2:**
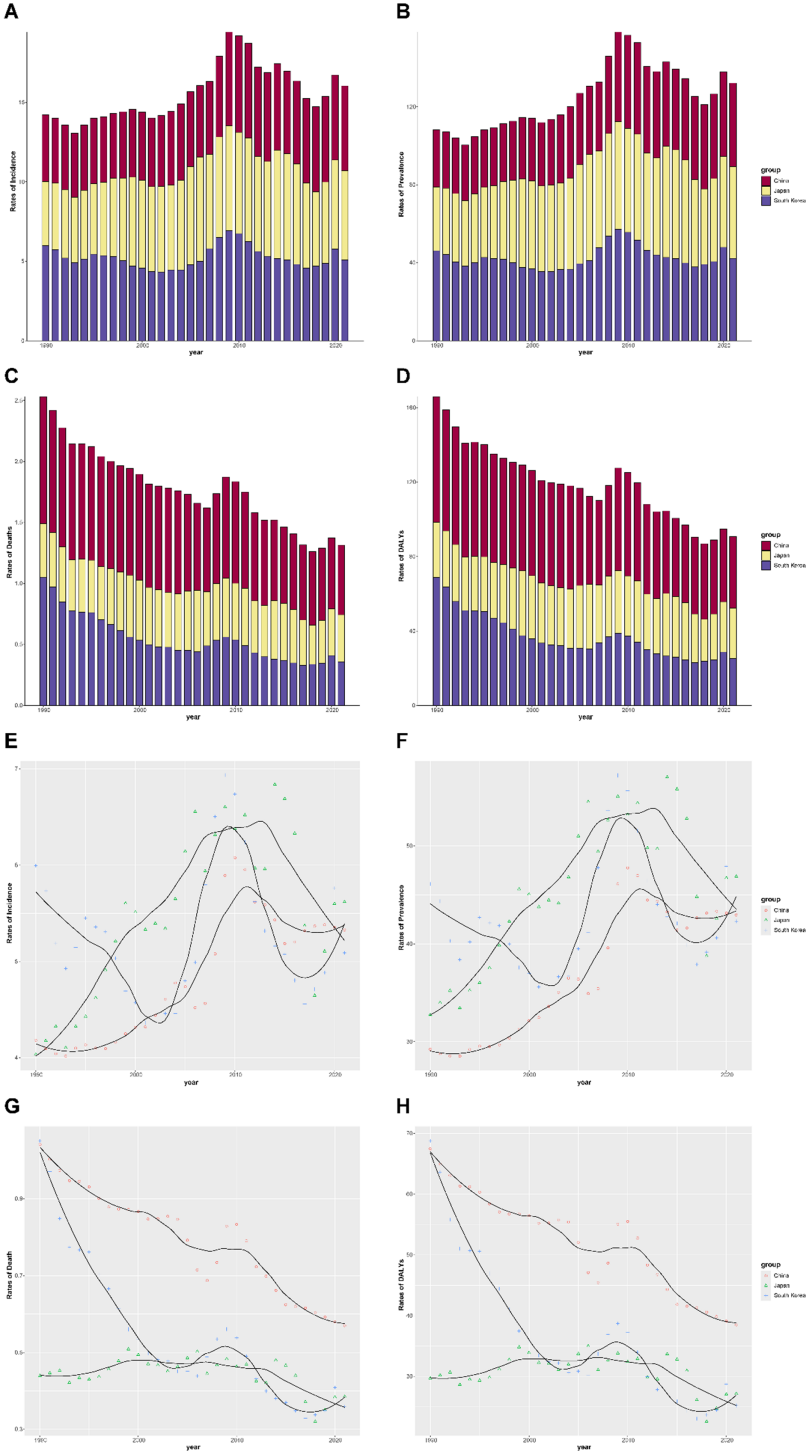
Temporal trends in cervical cancer burden among females under 40 years in China, Japan, and South Korea, 1990–2021. **(A)** Incidence rates, **(B)** prevalence rates, **(C)** mortality rates, and **(D)** DALYs stratified by country. Bars represent annual rates, with colors indicating different countries. **(E–H)** Fitted temporal trends of the same four indicators for China, Japan, and South Korea. Points represent observed values, and lines indicate smoothed estimates. The stacked bar charts and smoothed curves illustrate the temporal trends in cervical cancer burden. Bar heights represent the total burden, whereas colors indicate contributions from individual countries. The smoothed curves highlight the long-term changes. For example, Japan showed a steady rise in incidence and prevalence, whereas China and South Korea demonstrated declines in mortality and DALYs.

Figure 2 illustrates the temporal trends in cervical cancer burden among females under 40 years of age in the three East Asian countries from 1990 to 2021, revealing a complex pattern of change. Both China and Japan demonstrated an overall increasing burden across the study period for incidence and prevalence (Figures 2A,B,E,F), whereas South Korea remained relatively stable, with some fluctuations. In contrast, mortality and DALY rates (Figures 2C,D,G,H) declined markedly in China and South Korea, whereas Japan maintained consistently low and stable levels. Overall, the stacked bar charts show the annual contribution of each country to the total disease burden, while the smoothed curves highlight long-term disparities, most notably the pronounced increase in incidence and prevalence in Japan, alongside substantial reductions in mortality and DALYs in China and South Korea.

**Figure 3:**
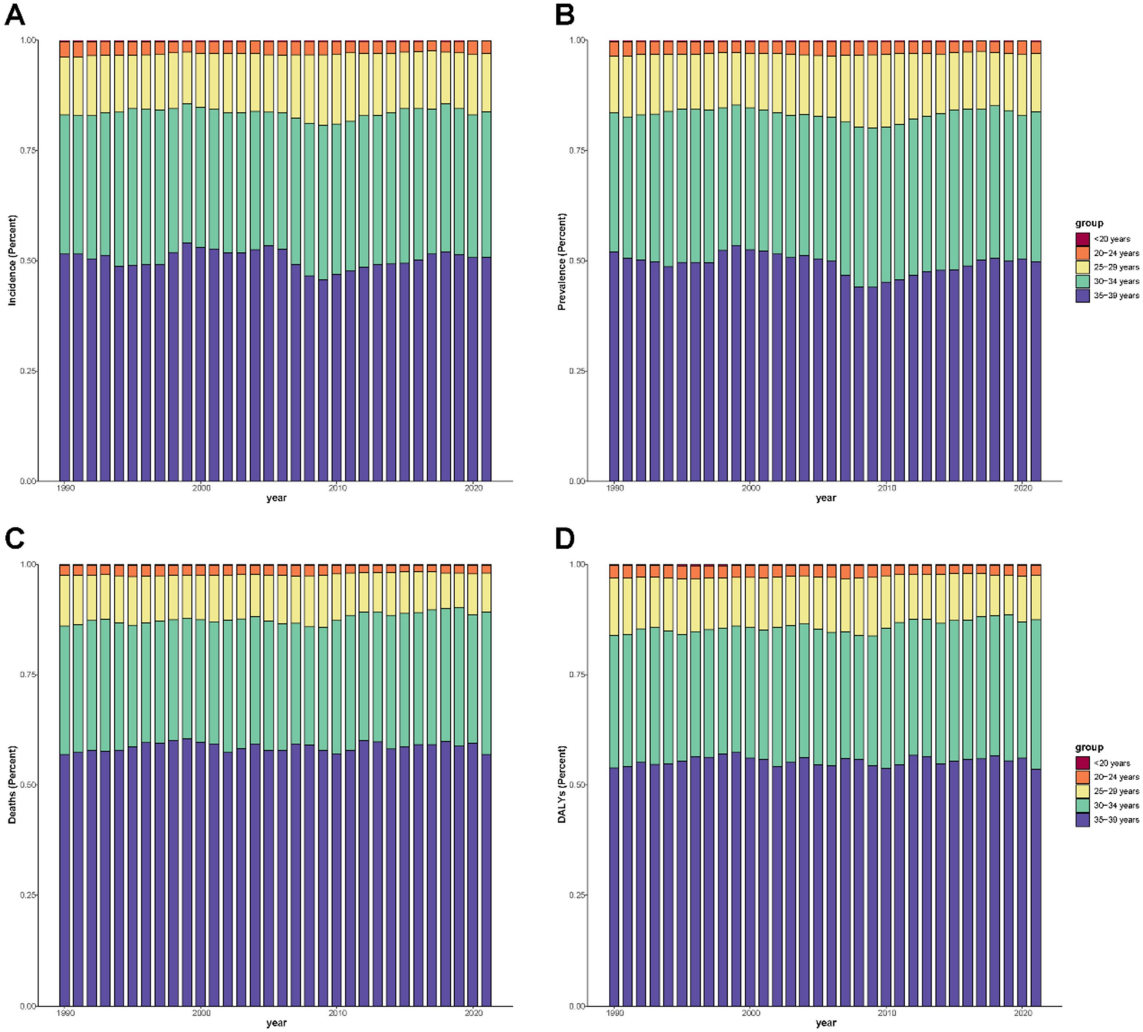
Age group composition of cervical cancer burden among females under 40 years old in China, Japan, and South Korea, 1990–2022. **(A)** Incidence, **(B)** prevalence, **(C)** mortality, and **(D)** DALYs contributed by each age group (<20, 20–24, 25–29, 30–34, and 35–39 years). Stacked bar charts display the yearly percentage contribution of each age group to the total burden, with colors indicating age categories.

Figure 3 presents the age group composition of the cervical cancer burden among females under 40 years in China, Japan, and South Korea from 1990 to 2021. Across all four indicators, the 35–39-year and 30–34-year groups consistently accounted for the largest share of the burden, followed by the 25–29-year group, while women under 20 years accounted for the smallest proportion throughout the study period. The overall age composition remained relatively stable over time in all three countries, although slight shifts were observed, including a marginal increase in the proportion attributable to the 35–39-year age group and a corresponding decline in younger age groups. These patterns were broadly consistent across China, Japan, and South Korea, indicating that the cervical cancer burden in East Asia is predominantly concentrated in women aged 30 years and older in the under-40 cohort.

## Discussion

This study provides a comprehensive overview of the cervical cancer burden among females under 40 years of age in East Asia, including China, Japan, and South Korea, using data from the GBD Study 2021, revealing distinct geographic and temporal trends in incidence, mortality, prevalence, and DALYs. Our findings show persistent and, in some cases, widening disparities between countries despite the preventable nature of cervical cancer and the availability of effective interventions.

Japan has maintained the highest incidence, prevalence, and DALY rates in 2021, with a steady upward trend in incidence across all age groups ≥20 years since 1990. This contrasts with the declining mortality and DALY rates in China and South Korea, where the incidence trends were either modestly increasing (China) or decreasing (South Korea). These patterns reflect the interplay between HPV vaccination, screening practices, and broader health system performance. Japan’s high incidence may partly result from improved detection through organized screening and better registry coverage; however, the concurrent increase in DALYs among older subgroups (≥30 years) suggests a true rise in disease occurrence that screening alone does not explain. The sharp decline in HPV vaccination coverage after the suspension of government recommendations in 2013 likely contributed to this trend.

China’s rising incidence in younger adult women, particularly those aged 25–34 years, combined with persistently high mortality, signals gaps in both primary and secondary prevention. HPV vaccines have only been available since 2016, with limited public funding and low coverage, and are often constrained by high out-of-pocket costs. Opportunistic screening remains predominant with variable quality and incomplete coverage, especially in rural areas. These structural barriers may have slowed progress compared to South Korea, which achieved higher vaccination rates following the 2016 inclusion of HPV vaccines in the national immunization schedule.

South Korea stands out for achieving concurrent declines in the incidence, mortality, and DALYs across most age groups. This likely reflects sustained investment in organized screening programs, high HPV vaccine uptake, and a strong public health infrastructure. Nonetheless, the persistence of the disease burden in women aged 30–39 years indicates that prevention efforts must also address older, previously unvaccinated cohorts.

Our results align with previous GBD-based analyses showing a negative correlation between the SDI and cervical cancer mortality and DALY rates (3). This study adds novel insights by focusing on women under 40 years of age and disaggregating trends across age strata. Previous studies have often grouped all reproductive-aged women together, obscuring important within-group differences. For instance, we observed a consistent shift in burden toward the 35–39-year age group, particularly in Japan and China, which may reflect both delayed HPV exposure in younger cohorts and insufficient vaccine protection among older women.

In Japan, prior research documented a rapid decline in HPV vaccination coverage from ~70% to below 1% following the 2013 policy suspension and the gradual recovery since recommendations resumed in 2021. Our analysis suggests that the public health impact of this gap is already observable in epidemiological trends, reinforcing the need for catch-up vaccination programs. Similarly, in China, modeling studies predict that without the substantial expansion of HPV vaccination and high-quality screening, cervical cancer incidence among younger women will continue to rise over the coming decades.

Although this analysis focused on women under 40 years of age, the direct age-disaggregated data for this subgroup are limited to the public GBD dataset. Nevertheless, indirect evidence suggests that the trends in the broader female population may not fully reflect the reality of younger women. For example, studies from Japan have shown a rising incidence of cervical cancer among women born in the 1980s and the 1990s, which is potentially linked to changes in sexual behavior, delayed marriage, and vaccination disruptions (11). Younger women may also face unique barriers to screening, such as lower perceived risk and limited access to gynecological services, particularly in rural or low-SDI areas. Given their reproductive potential and socioeconomic roles, failure to address cervical cancer in this group could result in substantial long-term health and economic costs. The age group composition reveals a slight shift in burden toward older age groups within the under-40 cohort. This may be influenced by delayed marriage and changes in sexual behavior, which alter HPV exposure patterns. Additionally, disparities in vaccination coverage across age groups may also contribute to these shifts.

Comparative findings from other regions highlight similar challenges in achieving WHO eliminating targets. For example, studies in Southeast Asia have also documented the impact of low HPV vaccination coverage on the cervical cancer burden. Our findings echo these concerns and emphasize the need for tailored strategies in East Asia to address the unique contextual factors that influence vaccination and screening uptake.

Primary prevention through HPV vaccination is widely recognized as the most effective strategy for reducing cervical cancer incidence (12). However, East Asian experience illustrates how policy implementation and public perception profoundly affect uptake. In China, HPV vaccines have been available since 2016 but are not included in the national immunization program, resulting in low coverage, estimated at only 4% for the first dose and 0.3% for full vaccination among eligible girls by 2022. In Japan, the suspension of government recommendations from 2013 to 2022 led to a dramatic drop in coverage, from around 70% to less than 1% (11). Although recommendations have resumed, recovery has been slow. These divergent experiences highlight the need for a strong political commitment, effective public communication, and integration of HPV vaccination into routine immunization schedules to ensure high and equitable coverage.

The WHO global strategy to eliminate cervical cancer as a public health problem aims for 90% of girls to be fully vaccinated by age 15, 70% of women screened with a high-performance test by ages 35 and 45, and 90% of women with precancer or cancer to receive treatment by 2030 (12). Our findings suggest that none of the three East Asian countries has met these targets for younger women, particularly in terms of vaccination coverage. Modeling studies indicate that the full implementation of the 90–70–90 strategy could eliminate cervical cancer in China by 2061, with earlier timelines possible in Japan and South Korea. Achieving this will require accelerated efforts in both primary and secondary prevention, with particular emphasis on reaching women under the age of 40.

### Strengths and limitations

This study leverages the standardized, comparable, and longitudinal GBD 2021 dataset to assess the cervical cancer burden over three decades across three major East Asian countries. By focusing on women under 40 years of age and stratifying by five-year age groups, we provide granular insights relevant to reproductive health policies.

However, this study has several limitations that warrant consideration. The GBD 2021 dataset provides valuable estimates of the disease burden. However, as a modeled dataset, it relies on input data of varying qualities. These estimates may be subject to bias in regions with incomplete cancer registry coverage. In addition, the data do not capture individual-level variations, which limits the granularity of our analysis. While this study leveraged the GBD 2021 dataset to provide valuable insights, several limitations must be acknowledged. As an ecological study, it is subject to an ecological fallacy, where conclusions drawn at the population level may not hold true at the individual level. Furthermore, this study’s reliance on modeled data limits our ability to establish causal relationships. The aggregated nature of the data also poses the risk of overinterpretation, particularly for the under-40 subgroup. Second, GBD estimates are model-based and rely on variable-quality input data, which may lead to underestimation or overestimation, especially in settings with incomplete cancer registry coverage. Third, the GBD dataset does not provide publicly available, fully disaggregated estimates for females under 40 years by individual country; therefore, we relied on the 15–39-year group as a proxy. Third, ecological correlations with the SDI and vaccination coverage cannot establish causality. Finally, risk factor attribution is based on modeled estimates and may not capture country-specific nuances in HPV genotype distribution or behavioral patterns. Last but not least, while temporal associations between HPV vaccination coverage and cervical cancer burden are discussed, these findings are ecological and should not be interpreted as causal. Without lag-time analyses or individual-level data controlling for behavioral or screening-related factors, causality cannot be established.

## Conclusion

Cervical cancer remains a substantial health challenge for women aged <40 years in East Asia, with divergent trajectories across China, Japan, and South Korea. The persistently high incidence in Japan, rising incidence and mortality in China, and the overall decline in South Korea reflect the cumulative impact of prevention policy decisions over the past three decades. Intensifying HPV vaccination, expanding screening, and targeting high-burden age groups are urgent priorities for achieving the WHO elimination goals and safeguarding reproductive health in the region. Our findings demonstrate the persistent burden of cervical cancer among females aged <40 years in East Asia. Divergent trends across China, Japan, and South Korea highlight the need for context-specific strategies to enhance HPV vaccination coverage and improve screening programs. While our empirical analysis provides a foundation for understanding current trends, it also reveals gaps that must be addressed to align with WHO’s elimination goals. Regional health authorities must prioritize these actions to safeguard reproductive health and reduce the impact of cervical cancer.

## Data Availability

The original contributions presented in the study are included in the article/supplementary material, further inquiries can be directed to the corresponding author/s. To download GBD data used in these analyses, please visit the Global Burden of Disease (GBD) website.

## Footnotes

^1^ https://ghdx.healthdata.org/gbd-results-tool

^2^ https://ghdx.healthdata.org/gbd-results-tool
